# Fracture resistance of teeth restored with polyethylene fibers reinforced composite restorations: a systematic review and meta-analysis of *in vitro* studies

**DOI:** 10.3389/fdmed.2025.1733879

**Published:** 2026-01-22

**Authors:** Alejandra Alvarado-Orozco, Rim Bourgi, Laura Emma Rodríguez-Vilchis, Carlos Enrique Cuevas-Suárez, Rosalía Contreras-Bulnes, J. Eliezer Zamarripa-Calderón, José Alejandro Rivera-Gonzaga

**Affiliations:** 1 Universidad Autónoma del Estado de México, Facultad de Odontología, Centro de Investigación y Estudios Avanzados en Odontología “Dr. Keisaburo Miyata” (CIEAO), Jesús Carranza esq. Paseo Tollocan, Col. Universidad, Toluca, Estado de México, México; 2 Department of Restorative and Esthetic Dentistry, Faculty of Dental Medicine, Saint-Joseph University of Beirut, Beirut, Lebanon; 3 Department of Biomaterials and Bioengineering, INSERM UMR_S 1121, University of Strasbourg, Strasbourg, France; 4 Department of Restorative Sciences, Faculty of Dentistry, Beirut Arab University, Beirut, Lebanon; 5 Dental Materials Laboratory, Academic Area of Dentistry, Autonomous University of Hidalgo State, San Agustín Tlaxiaca, México

**Keywords:** composite resin, fiber-reinforced composites, fracture resistance, *in vitro*, polyethylene fibers

## Abstract

**Aim:**

This systematic review and meta-analysis aimed to evaluate the fracture resistance of permanent teeth restored with polyethylene fiber-reinforced composite resin restoration.

**Material and methods:**

The following PICOS framework used was: Population, permanent teeth requiring restorative treatment; Intervention, polyethylene fiber use; Control, composite resin restorations without fiber reinforcement, or conventional fiber posts; Outcome, fracture resistance (in Newtons); Study design, *in vitro* studies. A literature search was conducted independently by two reviewers up to May 18, 2025, using electronic databases (PubMed, ISI Web of Science, SciELO, Scopus, and Embase). *In vitro* studies examining the fracture resistance of permanent teeth restored with polyethylene fiber-reinforced resin composite restorations were included. Meta analyses were performed by comparing the standardized mean differences in the fracture resistance of teeth restored using polyethylene fibers and the teeth restored only with resin composite. Additional analysis was performed comparing the risk difference of the number of unfavorable fractures. Separate analyses were performed when fiber posts were used. A *p*-value of less than 0.05 was considered statistically significant.

**Results:**

The fracture resistance of polyethylene fiber-reinforced restorations was higher compared to non-fiber reinforced composite restorations (*p* < 0.001). Also, the number of unfavorable fractures were significant lower when polyethylene fibers were used (*p* < 0.001).

**Conclusion:**

The findings suggest that the fracture resistance of permanent teeth may be improved with the use of polyethylene fibers. However, clinical performance outcomes are necessary to validate these *in vitro* results.

**Systematic Review Registration:**

The protocol for the systematic review was developed a priori and can be accessed at: https://doi.org/10.17605/OSF.IO/5K8XB.

## 1 Introduction

In restorative dentistry, a primary objective is to replicate the biomechanical performance of the natural dentition. This is particularly challenging when treating teeth with extensive coronal structure loss, where large cavity preparations or endodontic treatment further compromise structural integrity (1, 2). Composite resin is the material of choice for direct esthetic reconstructions due to its superior aesthetics and adhesive properties. However, when used in extensive restorations, its inherently brittle polymer matrix can lead to inadequate fracture resistance and catastrophic failure under functional occlusal loads (2).

To address these mechanical limitations, various fiber reinforcement strategies have been developed. Among them, polyethylene fibers have gained attention due to their unique properties, including high tensile strength, a low modulus of elasticity similar to dentin, and superior fracture toughness (3, 4). When incorporated into a resin composite matrix, these fibers are proposed to act as a stress-bridging network, improving impact resistance, distributing occlusal forces more uniformly, and reducing crack propagation (4–6). This mechanism holds promise for enhancing the longevity of large direct restorations and restorations involving fiber posts in endodontically treated teeth.

Despite the theoretical advantages, the *in vitro* evidence regarding the efficacy of polyethylene fiber reinforcement remains inconclusive. Several studies report a significant increase in fracture resistance for teeth restored with polyethylene fiber-reinforced composites or posts (7, 8), while others found no statistically significant difference compared to non-reinforced controls (9, 10). This discrepancy may be attributed to variations in study design, including fiber type, placement technique, cavity configuration, and testing protocols.

Given these inconsistent findings, a systematic evaluation and quantitative synthesis of the existing *in vitro* data are necessary to clarify the actual effect of polyethylene fiber reinforcement on fracture resistance. Therefore, this systematic review and meta-analysis aimed to assess whether the use of polyethylene fiber-reinforced composite resin or fiber post restorations improves the fracture resistance of permanent teeth compared to non-fiber-reinforced restorations. The study tested the null hypothesis that no significant difference exists between the fracture resistance values of teeth restored with and without polyethylene fiber reinforcement.

## 2 Materials and methods

The present study was conducted following the PRISMA (Preferred Reporting Items for Systematic reviews and Meta-Analyses) guidelines (11). The protocol was developed *a priori* and can be accessed at: https://doi.org/10.17605/OSF.IO/5K8XB. The following PICOS framework was applied: Population, permanent teeth requiring restorative treatment; Intervention, restoration with polyethylene fiber-reinforced composite resin or polyethylene fiber-reinforced fiber posts; Control, restoration with non-fiber-reinforced composite resin, or conventional fiber posts; Outcome, fracture resistance (in Newtons); Study design, *in vitro* studies. Eligible controls thus included any standard restorative alternative against which the polyethylene fiber-reinforced intervention was compared within a study. The research question was: Does the use of polyethylene fiber-reinforced composite restorations increase the fracture resistance of permanent teeth?

### 2.1 Literature search

The systematic search was conducted independently by two authors (A.A.-O. and R.B.) up to May 18th, 2025, without date restriction, among five electronic databases (PubMed, ISI Web of Science, SciELO, Scopus, and Embase). Table 1 lists the keywords and search strategies implemented in PubMed, which were then adapted for use in other databases. Additionally, the reviewers carried out a manual search of the reference lists from the included articles to find additional relevant literature. All identified articles from the databases were imported into Mendeley Desktop version 1.17.11 to eliminate duplicates.

**Table 1:** Search strategy used.

Database	Search strategy
PubMed	((Teeth restored OR posts and core systems OR endodontically-treated teeth OR dental post OR post-Core) AND (Ribbond OR polyethylene fiber OR fiber reinforced composite OR fiber reinforcement OR FRC – systems)) AND (Fracture resistance OR resistance to fracture)microshear bond strength OR shear bond strength OR performance)
Web of Science and Scielo	TS = (Teeth restored OR posts and core systems OR endodontically-treated teeth OR dental post OR post-Core) AND TS = (Ribbond OR polyethylene fiber OR fiber reinforced composite OR fiber reinforcement OR FRC – systems) AND TS = (Fracture resistance OR resistance to fracture)
SCOPUS	TITLE-ABS-KEY(“Teeth restored” OR “posts and core systems” OR “endodontically-treated teeth” OR “dental post” OR “post-Core”) AND TITLE-ABS-KEY(“Ribbond” OR “polyethylene fiber” OR “fiber reinforced composite” OR “fiber reinforcement” OR “FRC – systems”) AND TITLE-ABS-KEY(“Fracture resistance” OR “resistance to fracture”)
EMBASE	“Teeth restored” OR “posts and core systems” OR “endodontically-treated teeth” OR “dental post” OR “post-Core” AND “Ribbond” OR “polyethylene fiber” OR “fiber reinforced composite” OR “fiber reinforcement” OR “FRC – systems” AND “Fracture resistance” OR “resistance to fracture”

### 2.2 Study selection

Titles and abstracts were initially screened by two reviewers (A.A.-O. and R.C.-B.) in order to identify studies that potentially met the following eligibility criteria: (1) *in vitro* studies reporting the fracture resistance of permanent teeth restored with polyethylene fibers-reinforced composite resin or fiber post restorations; (2) studies including mean and standard deviation (SD) data in Newtons (N) on resistance to fracture; (3) studies published in English. Case reports, case series, pilot studies, and reviews were excluded from the initial review. All potentially relevant articles were thoroughly reviewed. If the title and abstract did not provide sufficient clarity for a decision, the article was selected for full-text analysis. Each full manuscript was independently assessed by two investigators in duplicate. Any discrepancies or differences regarding the suitability of the included articles were resolved through consultation with a third reviewer, who was a senior expert in the field (L.E.R.-V.). Only manuscripts that encountered the appropriateness criteria were integrated for review.

### 2.3 Data extraction

Data of interest from the comprised articles were inserted into standardized worksheets using Microsoft Office Excel 2021 software (Microsoft Corporation, Redmond, WA, USA). These data included the year of publication, author, polyethylene fibers used, substrate (cavity type or fiber post), composite resin used, outcomes evaluated (mean, SD, n), storing conditions, and type of failure. If any information was missing the corresponding author of the article was contacted to supply the exact data. If a response was not obtained within 2 weeks of the first contact, the missing information was not comprised.

### 2.4 Quality assessment

The methodological quality and risk of bias (RoB) of the included *in vitro* studies were assessed independently by two reviewers (A.A.-O. and J.E.Z.-C.) using a checklist adapted from a previous systematic review in dental materials research (12). This tool was selected as it is tailored to *in vitro* studies and evaluates key domains affecting internal validity, analogous to the principles of Cochrane RoB tools but adapted for preclinical testing (e.g., presence of a control group, sample randomization and standardization, blinding of the test operator, and appropriate statistical analysis). The checklist comprised the following nine parameters: Q1. Presence of control group. Q2. Sample randomization. Q3. Sample size calculation. Q4. Standardization of samples and material. Q5. Identical experimental conditions across groups. Q6. Adequate and standardized testing procedures and outcomes. Q7. Blinding of the test operator. Q8. Appropriate statistical analyses. Q9. Reporting study outcomes. Any disagreement in the RoB assessment between the two primary reviewers was resolved through discussion to reach a consensus. If a consensus could not be reached, a third senior reviewer (L.E.R.-V.) was consulted to make the final decision. Each parameter that was adequately described in the study received a “YES,” while omitted data received a “NO.” The overall risk of bias was classified according to the total number of “YES” responses: 1 or 2 indicated high bias, 3 to 5 indicated medium bias, and 6 or 7 indicated low risk of bias.

### 2.5 Statistical analysis

Meta-analyses were conducted using the Review Manager Software (version 5.4, The Cochrane Collaboration, Copenhagen, Denmark). A random-effects model was applied for the analyses, and pooled-effect estimates were calculated by comparing the standardized mean differences in the fracture resistance of teeth restored using polyethylene fibers and the teeth restored only with resin composite. Separate meta-analyses were conducted based on the type of restoration: one comparing polyethylene fiber-reinforced composite restorations to non-fiber-reinforced composite restorations, and another comparing polyethylene fiber-reinforced fiber posts to conventional fiber posts. This approach was taken to account for the different control groups used across the included studies. Additional analysis was performed comparing the risk difference of the number of unfavorable or catastrophic fractures after the fracture resistance tests. Separate analyses were performed when fiber posts were used. A *p*-value of less than 0.05 was considered statistically significant. Statistical heterogeneity among studies was evaluated using the Cochran Q test and the I^2^ inconsistency test. The potential for publication bias was assessed visually through funnel plots.

## 3 Results

**Figure 1:**
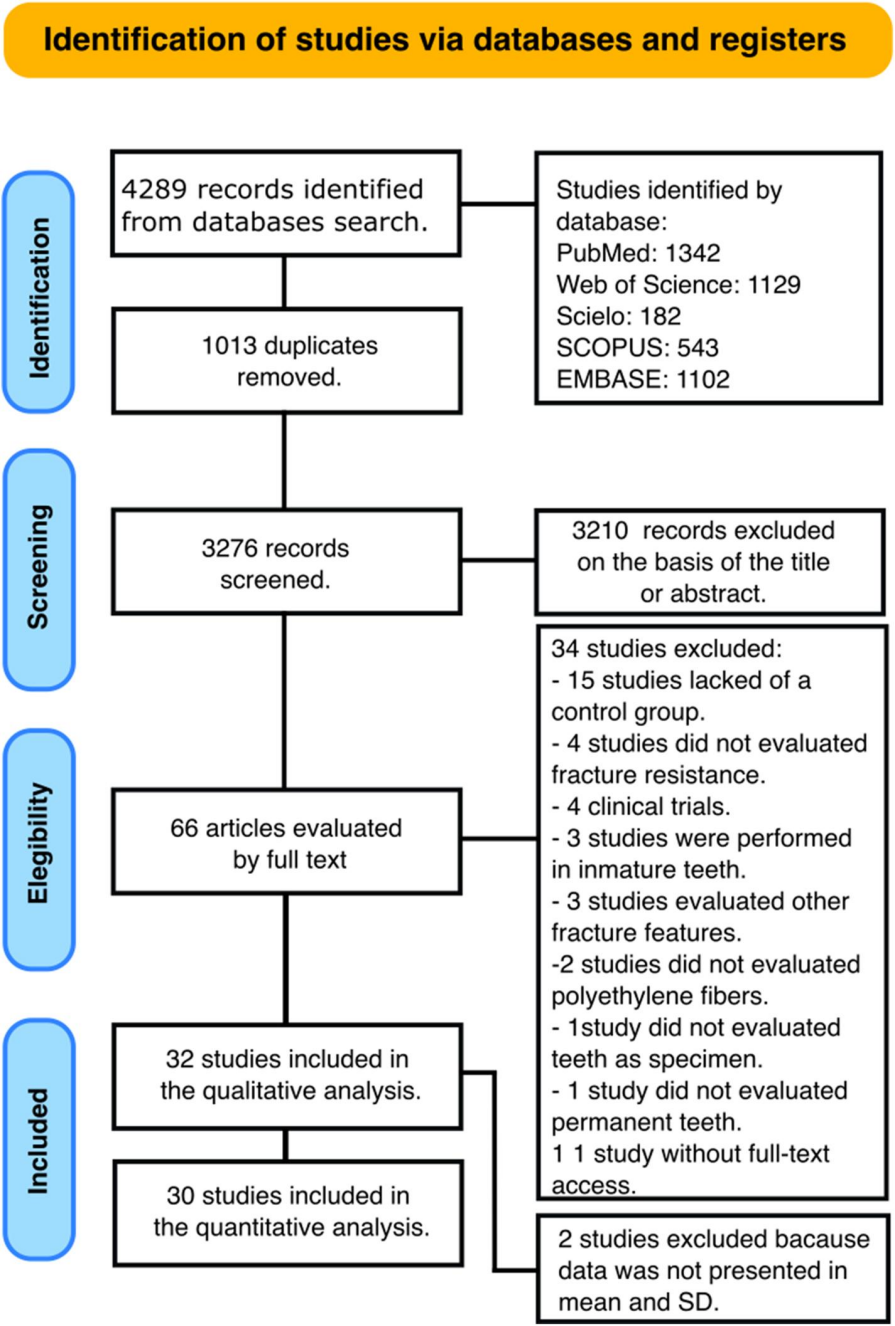
Flowchart summarizing the selection process.

A total of 4,289 papers were recognized in all databases searched. A flowchart that forms the report selection procedure agreeing to the PRISMA Statement is displayed in Figure 1. The literature review rescued 3,276 articles for the initial inspection after removing the duplicates. Afterward, 3,210 studies were excluded after reviewing the titles and abstracts, leaving 66 articles to be assessed by full-text interpretation. After the full-text assessment, 34 studies were excluded due to the following reasons: fifteen lacked a control group (13–27), four evaluated other physical properties different to fracture resistance (24, 26, 28, 29), four were clinical trials (30–33), three evaluated fracture resistance in permanent teeth with immature apices (34–36), three applied facture strength test to other points but not the teeth (16, 20, 37), two used another fiber type (38, 39), one did not use the teeth as specimens (40), one did not evaluate fracture resistance in permanent teeth (41), and one because access to the full document could not be obtained (42). The specific reason for each excluded study, mapped to the violated PICOS criterion, is provided in Supplementary Table S1.

A total of thirty-two manuscripts were included in the qualitative analysis, and from these, two were excluded from the meta-analysis because they did not have complete statistical data (43, 44). Finally, thirty manuscripts were included in the meta-analysis (3, 5, 22, 42, 45–70).

This review identified that the overwhelming majority of the included studies investigated the Ribbond™ brand (Ribbond Inc., Seattle, WA, USA) of polyethylene fibers. Thus, the findings of this synthesis pertain primarily to this specific commercial product applied alongside resin composite in Class I and II cavities of permanent teeth, with or without prior endodontic treatment. It also included fiber post restorations reinforced with Ribbond in teeth that underwent root canal treatment. The various composite resins and fiber posts were evaluated using fracture resistance tests, and the storage conditions of the samples included distilled water, saliva, chloramine solution, or thermocycling regimes for varying durations (Table 2). A detailed summary of the cavity configurations and fiber placement strategies employed in the included studies is also provided. It is noteworthy that all included studies assessed fracture resistance using a monotonic static loading protocol; no studies utilizing cyclic fatigue tests met the inclusion criteria for this review.

**Table 2:** Characteristics of the studies in the qualitative review.

Study	Substrate and cavity configuration	Aging protocol	Adhesive & Composite Resin used	Fiber Config./Orientation	Main results
Newman 2003	Endodontically treated maxillary central incisors with post space preparation.	100% humidity at temperature for 24 h	Scotchbond Multi-Purpose (3M ESPE) Cement-It (Pentron Clinical Technologies)	Cervical and middle third of the root canal	The load to failure of the stainless-steel posts were significantly stronger than all the composite posts
Belli 2005	MOD preparations on endodontically treated human mandibular molars.	100% humidity at 37 °C for 1 day	Clearfil SE Bond (Kuraray) Clearfil AP-X (Kuraray)	Cervical third of the buccal- lingual wall	Use of polyethylene ribbon fiber under composite restorations increased fracture strength.
Belli 2006	MOD preparations on endodontically treated human mandibular molars.	100% humidity at 37 °C for 24 h	Clearfil SE Bond (Kuraray) Clearfil AP-X (Kuraray)	Occlusal and cervical third of the buccal to lingual wall	Polyethylene fiber use over or under MOD composite restorations significantly increased fracture strength.
Belli 2006 (b)	MOD preparations on endodontically treated human mandibular molars.	100% humidity for 24 h	Clearfil SE Bond (Kuraray) Clearfil AP-X (Kuraray)	Cervical and middle third of the buccal- lingual walls	The insertion of Ribbond inside the cavity has a positive effect on frture strength of endodontically treated molar teeth with MOD cavity preparation and cuspal fracture.
Cobankara 2008	MOD preparations on endodontically treated human mandibular molars.	100% humidity at 37 °C for 1 day	Clearfil SE Bond (Kuraray) Clearfil Photoposterior (Kuraray)	Cervical third of the buccal- lingual wall	Indirect hybrid inlay is more favorable fracture failure modes than other restoration techniques
Segun 2008	MOD preparations on endodontically treated human mandibular premolars.	100% humidity at 37 °C for 24 h	Clearfil SE Bond (Kuraray) Clearfil AP-X (Kuraray)	Cervical and middle third of the buccal- lingual walls	A combination of polyethylene fiber and composite resin were not significantly different than those that were restored with only composite resin
Ayad 2009	Class I and II preparation on mandibular molars.	Distilled water at room temperature for 7 days	Optibond Solo Plus (Kerr) Prodigy (Kerr)	The floor of the cavity	Class I cavities with fiber-reinforced resin composite had the highest fracture strength. Class II cavities restored with fiber-reinforced resin composite had intermediate fracture strength
Badakar 2011	Class IV on endodontically treated human maxillary central incisors	1% chloramine solution for 24 h	Esthet X (Dentsply/Caulk) Ceram X (Dentsply/Caulk)	The cavity as close to enamel	Fibre-reinforced composite used for the restoration of fractured incisal edge has achieved the fracture resistance almost equal to intact natural tooth.
Kalburge 2013	MOD preparation on endodontically treated human maxillary premolars	Thermocycling of 500 cycles between 5 °C and 55 °C water baths	Adper single Bond (3M ESPE) Filtek Z-250 (3M ESPE)	Buccal to lingual direction in floor of the cavity	A composite restored and Ribbond reinforced composite restored maxillary premolars can help preserve the fracture resistance of teeth
Khan 2013	MOD preparation on endodontically treated human mandibular molars	100% humidity at 37 °C for 1 day	Gluma (Heraeus, Kulzer) Venus (Heraeus, Kulzer)	The buccal wall, pulpal floor and lingual wall	Teeth restored with polyethylene and glass fibers showed increased fracture resistance
Kumar 2013	Endodontically treated mandibular premolars with post space preparation.	Moist environment for 1 week	RelyX U100 (3M ESPE)	Cervical and middle third of the root canal	Vertically fractured teeth can be treated by filling the root canal space with dual-cure adhesive resin cement or by adding polyethylene fiber or glass fiber to increase the fracture resistance of the reattached tooth fragments
Costa 2014	MOD preparation on endodontically treated human maxillary premolars	Thermocycling 1,000 times between 5 °C and 55 °C	Single Bond (3M ESPE) Filtek Z250 (3M ESPE)	Into the cavity	Ribbon–fiber reinforced resin restorations provided superior fracture resistance of premolars
Braga 2015	MOD preparation on endodontically treated human maxillary premolars	After 24 h	Adper single Bond (3M ESPE) Filtek Z-250 (3M ESPE)	Into the root canal	Premolars restored with polyfiber post (Spirapost), regardless of the composite resin (only microhybrid or microhybrid combined with flowable) provided the highest fracture strength
Miao 2016	MOD preparation on endodontically treated human maxillary premolars	37 °C in 100% humidity for 24 h	Single Bond (3M ESPE) Filtek P60 (3M ESPE)	Buccal to lingual direction	Polyethylene fiber reinforced composite restorations strengthened the fracture resistance
Garlapati 2017	MOD preparation on endodontically treated human mandibular molars	Thermocycling as recommended by Gale and Darvell: 35 °C (28s), 15 °C (2s), 35 °C (28s), 45 °C (2s) w	Te-Econom Bond (Ivoclar Vivaden) Te-Econom Plus, (Ivoclar Vivadent)	Pulpal floor, buccal and lingual walls	Teeth restored with everX posterior fiber reinforced composite showed superior fracture resistance.
Hshad 2017	MOD preparation on endodontically treated human mandibular premolars	Unmentioned	Clearfil SE Bond (Kuraray) Clearfil AP-X (Kuraray)	Buccal, lingual walls and pulpal floor	Use of reinforced polyethylene ribbon fiber beneath composite restorations in root-filled premolar teeth with MOD preparations considerably increased the fracture strength.
Aslan 2018	MOD preparation on endodontically treated human mandibular premolars	Distilled water at 37 °C for 24 h	Single Bond Universal (3M ESPE) Filtek Ultimate (3M ESPE)	Buccal, lingual walls and pulpal floor	Usage of horizontal post or occlusal Ribbond usage increased the fracture resistance of root canal-treated premolars with MOD cavities.
Khan 2018	MOD preparation on endodontically treated human mandibular molars	Incubator at 37 °C in 100% humidity for 24 h	Te-Econom Bond (Ivoclar Vivaden) Te-Econom Plus, (Ivoclar Vivadent)	Buccal, lingual walls and pulpal floor	Everstick and Bioctris showed higher fracture resistance when compared to Ribbond and Dentapreg
Eliguzeloglu 2019	MOD preparation on endodontically treated human mandibular premolars	Unmentioned	Clearfil SE Bond (Kuraray) Estelite Bulk Fill Flow (Tokuyama)	Pulpal floor, 2/3 buccal and lingual walls and/or in half of the cavity	Fiber insertion with different techniques did not increase the fracture strength of teeth restored with bulkfill composites, it increased the favorable fracture modes. Thermomechanical aging did not change the fracture strength of the groups
Fildisi 2022	Overlay or endocrown preparation on endodontically treated human mandibular molars	Thermocycling at 10,000 cycles at 5 °C and 55 °C	Clearfil SE Bond (Kuraray) Gradia Direct Posterior (GC)	Pulpal floor, buccal and lingual walls	Overlay restorations showed higher fracture strength values than endocrown restorations. Ribbond did not significantly change the fracture strength values of the endocrown and overlay restorations but reduced the frequency of irreparable fracture modes
Sreen 2022	Incisal fracture on central incisors.	Unmentioned	Bonding agent (Ivoclar Vivadent) Hybrid composite (Ivoclar Vivadent)	Vertical grooves	The highest values for force required to fracture were observed in the fiber post group and the lowest in the Ribbond group
Albar 2023	MOD preparation on molars	Thermocycling for 10,000 cycles at 5 °C and 55 °C for 24 h	Filtek Z250 (3M ESPE)	Axial walls and the floor of the cavit	EverX Posterior had a statistically significant higher maximum load resistance versus the control and versus the polyethylene fibers
Soto-Cadena 2023	MOD preparation on endodontically treated human maxillary premolars	Unmentioned	Optibond FL (Kerr) IPS Empress Direct (Ivoclar)	Pulpal floor, lingual and buccal wall	Reinforcing endodontically treated premolars with MOD cavities with Ribbond fibers followed by a conventional composite resin enhanced fracture resistance The effect of placing ever X over Ribbond fibers in MOD cavities in endodontically treated premolars increased fracture resistance values.
Denger 2023	MOD preparation on human maxillary premolars	Distilled water for 24 h	Clearfil Universal Bond Quick (Kuraray) Filtek Z250 (3M ESPE)	Into the cavity	The use of polyethylene fiber exhibits significantly greater gap formation
Tsertsidou 2023	MOD preparation on endodontically treated human mandibular molars.	Thermocycling of 10,000 cycles in deionized water solution at 5 and 55 °C	Adhese Universal (Ivoclar Vivadent) Tetric, (Ivoclar Vivadent)	Pieces measuring 3 mm and 2 mm in thickness and were used as a reinforcement under the resin composite with 1 mm of thickness	CAD/CAM inlays and fiber-reinforced composites offer improved fracture resistance
Albashaireh 2024	Endodontically treated maxillary central incisors with post space preparation.	Thermocycling for 5,000 cycles in distilled water at 5 and 55 °◦C	Clearfil SE Bond (Kuraray) Clearfil AP-X (Kuraray)	Into the root canal	Fibre-reinforced composite cores significantly increased fracture resistance in endodontically treated anterior teeth with ferrule
Aljarboua 2024	MOD preparation on human premolars	Unmentioned	Single Bond Universal Adhesive (3M ESPE) Filtek Z250 XT (3M ESPE)	The pulpal floor only or proximal walls only or pulpal floor and proximal walls	That placing Ribbond fibers in the pulpal floor or the proximal wall resulted in improved fracture resistance. The combination of placing the Ribbond fiber in these two locations demonstrated enhanced strength exclusively in endodontically treated teeth.
Mohammadipour 2025	MOD preparation on human third molars.	Distilled water for 24 h at room temperature	Adper Single Bond 2 (3M ESPE) G-ænial Posterior (CG)	Pulpal floor, the first fiber was placed in the buccolingual direction and second fiber in the mesiodistal direction or only pulpal floor the buccolingual direction	The reconstruction with Ever X posterior or incorporating of Ribbond Ultra-THMin crosssectional direction on base of resin composite restoration improved fracture strength and favorably affected fracture modes in comparison to conventional posterior resin composite with or without cusp coverage.
Pierce 2025	MOD preparation on human third molars.	Thermocycling in distilled water for 2,000 cycles at 5 and 55 °C	Clearfil SE Bond (Kuraray) Filtek Supreme Ultra (3M ESPE)	Two pieces of polyethylene fiber the tooth surface in a crisscross pattern	The micrometer-scale, short-fiber-reinforced composite and nanofill composite group had the highest fracture load and was significantly greater than all other groups
Yüksek 2025	MOD preparation on endodontically treated human molars.	An incubator for 24 h at 37 °C	Scotchbond Universal Adhesive (3M ESPE) Filtek One Bulk-Fill (3 M ESPE)	Buccolingually in the middle third	The reinforcement of the coronal structure with fiber reduced cusp deflection and increased fracture strength.
Kaynar 2025	MOD preparation on endodontically treated human maxillary premolars.	Thermocycling 2,500 cycles 5 and 55 °C	Tokuyama Bond Force II (Tokuyama Dental) Tokuyama Estelite Posterior (Tokuyama Dental)	Pulpal floor to the 2/3 mm of the buccal and lingual walls	The highest fracture resistance was recorded in the groups restored with fibers.

**Figure 2:**
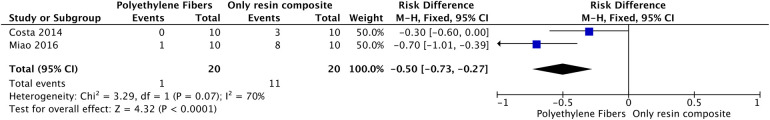
Meta-analysis of the fracture resistance of teeth restored with polyethylene fibers as intraradicular post.

Figure 2 shows the meta-analysis of the fracture resistance values of the polyethylene fibers reinforced fiber post. According to the analysis, fracture resistance was enhanced when the polyethylene fibers system was used (*p* < 0.01). A high heterogenicity (93%) was observed. The pooled Standardized Mean Difference (SMD) was 0.76 (95% CI: [1.17, 0.36), representing a moderate-to-large effect size according to Cohen's criteria (SMD > 0.8).

**Figure 3:**
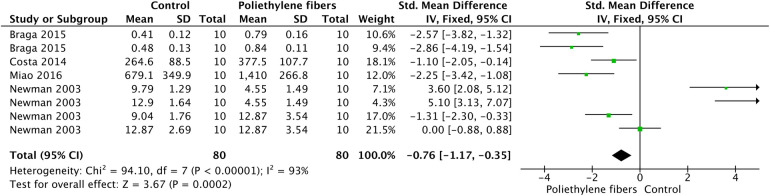
Meta-analysis of the fracture resistance of teeth restored with polyethylene fibers reinforced composite restoration.

Figure 3 shows the meta-analysis of the fracture resistance values of the polyethylene fibers reinforced composite resin restorations. According to the analysis, fracture resistance was enhanced when the polyethylene fibers system was used (*p* < 0.01). A high heterogenicity (89%) was observed. The pooled SMD was −0.82 (95% CI: [−1.16, −0.49]), representing a large effect size.

**Figure 4:**
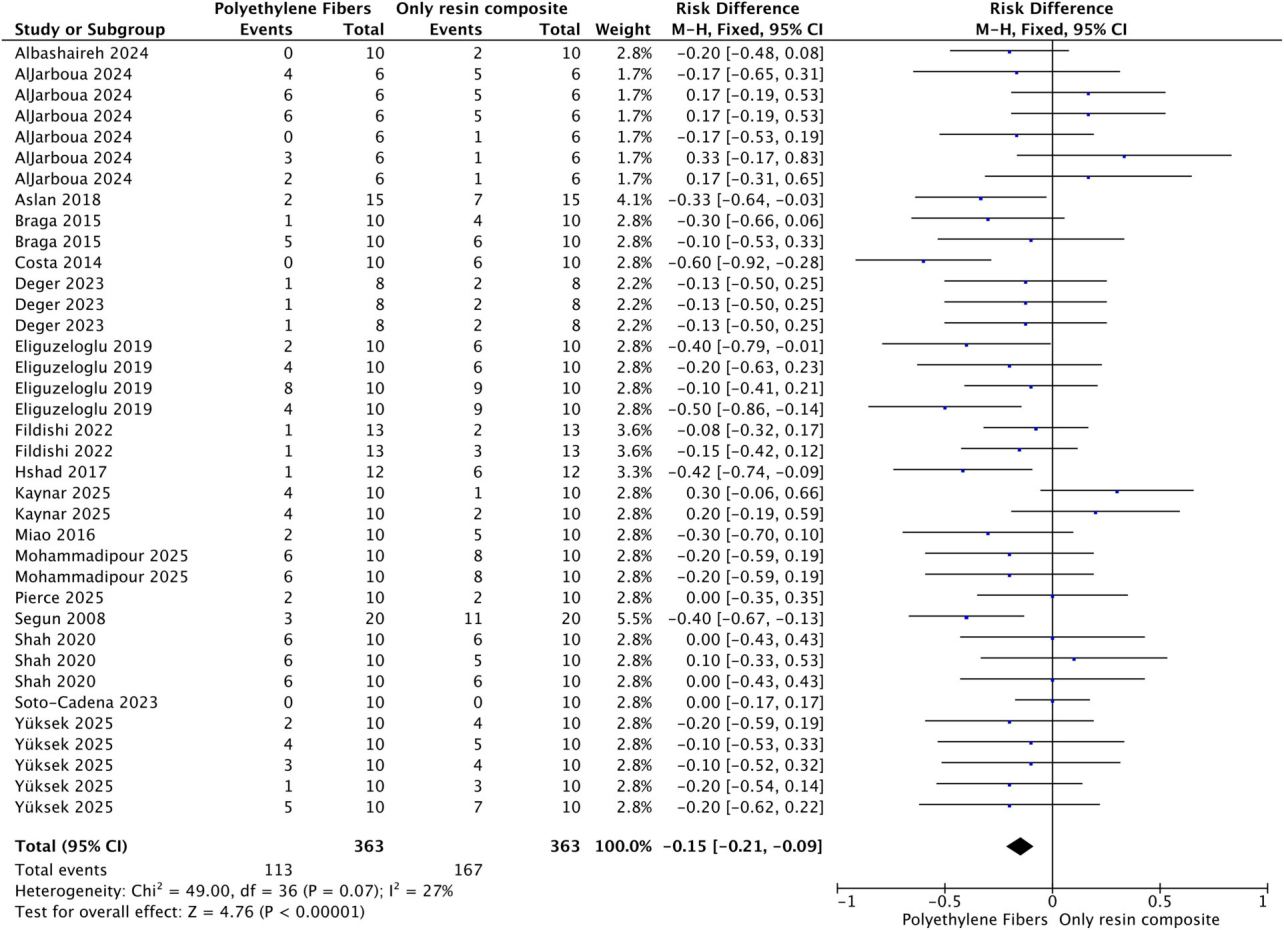
Meta-analysis of the number of unfavorable/catastrophic failures after the fracture strength test of teeth restored with polyethylene fibers used as intraradicular post.

**Figure 5:**
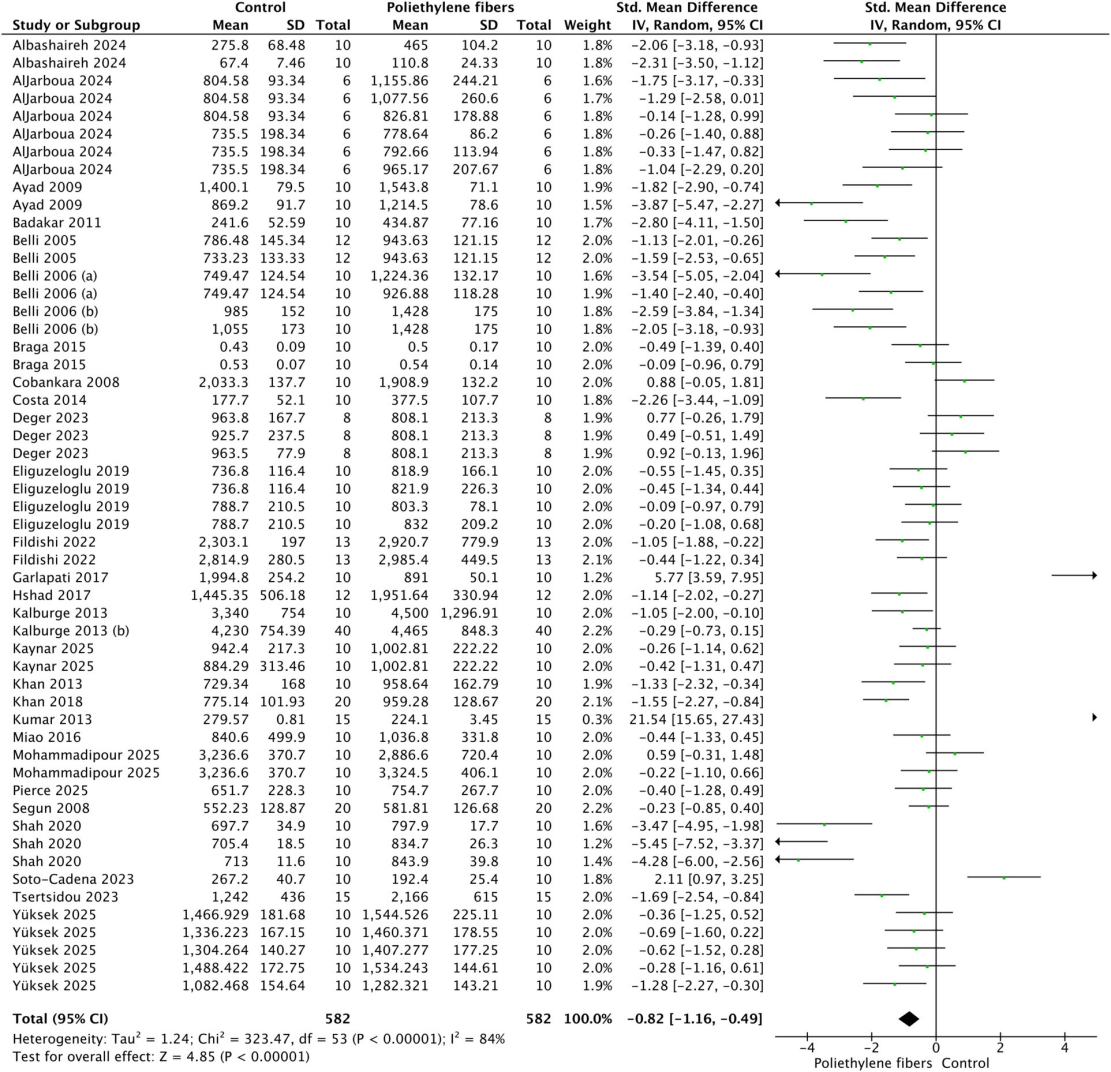
Meta-analysis of the number of unfavorable/catastrophic failures after the fracture strength test of teeth restored with polyethylene fibers reinforced composite restoration.

Figures 4, 5 show the meta-analysis of the number of unfavorable or catastrophic failures identified after the fracture resistance test. For both situations (with or without fiber posts), the use polyethylene fibers reduced the number of catastrophic failures in the restored teeth (*p* < 0.001). It is important to note that the specific definitions for “catastrophic” vs. “favorable” varied across studies, and no standardized re-classification was possible.

The high statistical heterogeneity observed in the meta-analyses reflects considerable clinical and methodological diversity across the included *in vitro* studies. Qualitative examination suggests potential sources include: the type of cavity preparation, the amount of remaining tooth structure, variations in the placement and orientation of polyethylene fibers, the use of different commercial brands of composite resins and adhesive systems and differing aging protocols.

The potential for publication bias in the meta-analyses was assessed through visual inspection of funnel plots. For the analysis of fracture resistance in teeth restored with polyethylene fiber-reinforced fiber posts (Figure 2), the funnel plot (Supplementary Supplementary Figure S2) showed a relatively symmetric dispersion of study estimates around the pooled effect size (SMD), with points scattered within the expected inverted funnel shape. This symmetry suggests a low likelihood of significant publication bias for this outcome.

In contrast, the funnel plot for the analysis of fracture resistance in teeth restored with polyethylene fiber-reinforced composite restorations (Figure 3, Supplementary Figure S3) revealed a more asymmetrical distribution. A noticeable gap was observed in the lower-left quadrant, indicating a potential absence of smaller studies reporting negative or null effects (i.e., no benefit from fiber reinforcement). While the heterogeneity (*I*^2^ = 89%) precludes definitive conclusions, this asymmetry hints at the possibility of publication bias, where smaller studies with non-significant results might be missing from the literature.

Regarding the analysis of catastrophic failures, both funnel plots exhibited high symmetry. The plot for the comparison involving fiber posts (Figure 4, Supplementary Figure S4) and the plot for direct composite restorations (Figure 5, Supplementary Figure S5) displayed a balanced distribution of studies on both sides of the pooled risk difference (RD) line. This consistent symmetry across both analyses strengthens the robustness of the finding that polyethylene fiber reinforcement significantly reduces the incidence of unfavorable fractures, as it is less likely to be driven by unpublished data.

The risk of bias in the articles studied in this review is recapped in Table 3. Most of the articles included failed to meet the parameters of blinded operator and sample size calculation.

**Table 3:** Risk of bias assessment.

Author	Q1	Q2	Q3	Q4	Q5	Q6	Q7	Q8	Q9
Newman 2003	YES	NO	NO	YES	YES	YES	NO	YES	YES
Belli 2005	YES	YES	NO	YES	YES	YES	NO	YES	YES
Belli 2006	YES	YES	NO	YES	YES	YES	NO	YES	YES
Belli 2006 (b)	YES	YES	NO	YES	YES	YES	NO	YES	YES
Cobankara 2008	YES	YES	NO	YES	YES	YES	YES	YES	YES
Segun 2008	YES	YES	NO	YES	YES	YES	YES	YES	YES
Ayad 2009	YES	YES	NO	YES	YES	YES	NO	YES	YES
Badakar 2011	YES	YES	NO	YES	YES	YES	NO	YES	YES
Kalburge 2013	YES	NO	NO	YES	YES	YES	NO	YES	YES
Khan 2013	YES	YES	NO	YES	YES	YES	NO	YES	YES
Kumar 2013	YES	YES	NO	YES	YES	YES	NO	YES	YES
Costa 2014	YES	YES	NO	YES	YES	YES	NO	YES	YES
Braga 2015	YES	YES	NO	YES	YES	YES	NO	YES	YES
Miao 2016	YES	YES	NO	YES	YES	YES	NO	YES	YES
Garlapati 2017	YES	YES	NO	YES	YES	YES	NO	YES	YES
Hshad 2017	YES	YES	NO	YES	YES	YES	NO	YES	YES
Aslan 2018	YES	YES	NO	YES	YES	YES	YES	YES	YES
Khan 2018	YES	NO	NO	YES	YES	YES	NO	YES	YES
Eliguzeloglu 2019	YES	YES	NO	YES	YES	YES	NO	YES	YES
Fildishi 2022	YES	YES	YES	YES	YES	YES	NO	YES	YES
Sreen 2022	YES	YES	NO	YES	YES	YES	NO	YES	YES
Albar 2023	YES	YES	YES	YES	YES	YES	NO	YES	YES
Soto-Cadena 2023	YES	NO	NO	YES	YES	YES	YES	YES	YES
Denger 2023	YES	YES	NO	YES	YES	YES	YES	YES	YES
Tsertsidou 2023	YES	YES	NO	YES	YES	YES	NO	YES	YES
Albashaireh 2024	YES	YES	YES	YES	YES	YES	YES	YES	YES
Aljarboua 2024	YES	YES	YES	YES	YES	YES	NO	YES	YES
Mohammadipour 2025	YES	YES	YES	YES	YES	YES	NO	YES	YES
Pierce 2025	YES	NO	NO	YES	YES	YES	NO	YES	YES
Yüksek 2025	YES	YES	YES	YES	YES	YES	NO	YES	YES
Kaynar 2025	YES	YES	YES	YES	YES	YES	NO	YES	YES

Q1. Presence of control group. Q2. Sample randomization. Q3. Sample size calculation. Q4. Standardization of samples and material. Q5. Identical experimental conditions across groups. Q6. Adequate and standardized testing procedures and outcomes. Q7. Blinding of the test operator. Q8. Appropriate statistical analyses. Q9. Reporting study outcomes.

## 4 Discussion

This systematic review and meta-analysis were directed to assess the fracture resistance of permanent teeth restored with polyethylene fiber-reinforced composite resin or fiber post restorations. The overall findings revealed that the polyethylene fibers in conjunction with composite resin or fiber post favored the fracture resistance of permanent teeth. Based on the findings of this study, the null hypothesis was rejected, indicating that the fracture resistance of permanent teeth with polyethylene fiber-reinforced composite restorations (*p* < 0.01) and polyethylene fiber-reinforced fiber posts (*p* < 0.01) significantly differs from that of non-fiber-reinforced composite and fiber post restorations. The large positive effect sizes (SMD > 0.8) indicate that the improvement in fracture resistance is not only statistically significant but also clinically substantial.

Ribbond fibers are composed of longitudinally oriented and crystallized polyethylene fibers. These fibers are used in combination with composite resin to reinforce teeth that have lost significant coronal structure. When applied adhesively, Ribbond fibers help enhance the mechanical properties of the tooth, increasing its strength and resistance to fracture by distributing forces uniformly and preventing crack propagation (48, 51, 71, 72). Also, the polyethylene fibers have been used to reinforce the fiber post in endodontically treated teeth to increase the durability and distribute forces along the teeth (51, 72). There are several studies evaluating the fracture resistance of teeth with polyethylene fiber-reinforced restorations (5, 45, 72). The mechanism of polyethylene fibers is to transfer stress from the polymer matrix of the resin composite into the fibers, which causes less stress to be transmitted to the remaining tooth structure (71).

In general, the studies included in this systematic review and meta-analysis indicate that fracture resistance improves with the use of polyethylene fibers. However, this improvement is influenced by other factors, such as the amount of remaining crown structure, the type of cavity preparation, and the arrangement or orientation of the fibers within the restoration. These variables play a significant role in determining the overall effectiveness of fiber-reinforced restorations in enhancing the tooth's mechanical stability (5).

The theoretical rationale for using polyethylene fibers is their potential to reinforce composite resin by improving stress distribution and bridging microcracks, thereby enhancing fracture resistance (47). However, the empirical evidence synthesized in this review reveals that this theoretical benefit is **highly contingent on technical execution**. While many studies report significant improvements (5, 48, 57), others—particularly those where fiber placement, orientation, or impregnation may have been suboptimal—found no significant difference or even a decrease in performance (60, 62). This dichotomy provides a compelling explanation for the high statistical heterogeneity observed (*I*^2^ > 89%). It suggests that the fibers' **high tensile strength and low modulus are advantageous only when they are correctly integrated** to absorb and distribute occlusal forces. Improper placement, such as positioning fibers in compression zones rather than tension zones, or poor adhesion creating voids, could inadvertently create stress concentrators, explaining the diminished performance in some studies.

Unlike conventional composite materials, which may exhibit brittle failure under high loads, polyethylene fibers introduce a toughening mechanism by bridging microcracks and dissipating stress along their length (49). Their high tensile strength and low-density modulus allow them to absorb and distribute occlusal forces more effectively, reducing the risk of catastrophic fractures (43). Additionally, their ability to conform to cavity walls and integrate within the composite matrix contributes to a more uniform load distribution, further enhancing the mechanical stability of the restoration (56).

Some studies have reported that the way in which polyethylene fibers are placed is related to fracture resistance. The most common way of placing them in mesio-occluso-distal (MOD) cavities is on the buccal, lingual/palatal wall and on the floor of the cavity (47, 54); however, two studies positioned the fibers on the occlusal surface and found that extending the fiber ends through the occlusal third of the buccal or lingual/palatal walls significantly enhanced fracture resistance. This strategic placement allowed the fibers to bind the cusps together, improving the structural stability of the restoration and enabling better distribution of forces during occlusion, ultimately leading to higher resistance against fractures (49). Regarding type of cavity, a study showed that Class I cavity preparation restored with fiber-reinforced resin composite was stronger than Class II cavities, due to the marginal ridges (49); Supporting this, fracture resistance is lower in MOD cavities restored with polyethylene fiber-reinforced composite resin compared to occluso-distal (OD) cavities, due to the loss of both marginal ridges (43, 68).

In this sense, it is important to highlight the fact that the position of polyethylene fibers is crucial for optimizing their reinforcing effect in composite restorations. For fibers to effectively absorb and distribute occlusal forces, they should ideally be placed on the opposite side of the applied stress, allowing them to stretch and resist tension (66). If fibers are positioned in areas where compressive forces prevail, their reinforcing function may be compromised. This emphasizes the need for careful consideration of fiber orientation in clinical applications to ensure the maximum fracture resistance of the restoration.

Other studies have reported that in teeth with root canal treatment, fracture resistance increases when they are reconstructed with polyethylene fiber reinforcements in conjunction with composite resin or fiber post, even when an extracoronal restoration such as an overlay or endocrown is placed after this (3, 56).

Research on tooth restoration using various fiber reinforcement materials, including long polyethylene fibers (Ribbond), short glass-fibers (EverX Posterior), and combinations of fibers, has yielded intriguing results. One study found that fracture resistance with polyethylene fibers was lower than with EverX Posterior (53, 56, 58). Conversely, another study reported that Ribbond outperformed EverX Posterior (73). Additionally, a third study indicated that when EverX Posterior was combined with Ribbond in MOD cavities, it resulted in an increase in fracture resistance values. These findings highlight the variability in outcomes depending on the type of fibers and how they are used in restorative procedures (57).

On the other hand, few studies have concluded that there is no significant difference in the fracture resistance of teeth restored with composite resins reinforced with polyethylene fibers compared to those restored only with composite resins (56, 58), however they also do not report that the fracture resistance is lower or affected when polyethylene fibers have been used, so more studies that include all the variables are necessary to have a clearer result regarding fracture resistance.

Beyond improving fracture resistance, the present meta-analysis also revealed that polyethylene fibers significantly reduced the number of unfavorable or catastrophic failures, both in teeth restored with and without fiber posts (*p* < 0.001). This finding suggests that the inclusion of polyethylene fibers not only enhances the strength of restorations but also influences the failure pattern, favoring more favorable, repairable fractures. Similar outcomes have been reported by previous *in vitro* studies, where the incorporation of Ribbond fibers into resin composite restorations promoted a shift from catastrophic root fractures toward restorable failures confined to the coronal structure (68–70). This effect has been attributed to the fibers' ability to absorb and redistribute stresses throughout the resin matrix, preventing stress concentration at critical points of the tooth-restoration interface (71, 72).

Therefore, the present findings should not be interpreted as an unconditional recommendation for polyethylene fiber use. Instead, they highlight a **technique-dependent intervention**. The consistent positive *directional* effect across the meta-analysis indicates a robust underlying potential for reinforcement. Yet, the inclusion of studies with null or negative results within that same analysis serves as a critical caveat: **realizing this potential in clinical practice requires meticulous attention to protocol**. Future research should move beyond asking *if* fibers work, to defining *how* they must be used—delineating the specific placement techniques, material combinations, and clinical indications that reliably unlock their reinforcing benefit while avoiding the pitfalls that lead to ineffective outcomes.

The reduction in catastrophic failures is particularly relevant in endodontically treated teeth, where the remaining dentin is structurally weakened and more susceptible to unfavorable fracture patterns. Polyethylene fibers act as a mechanical interlocking network within the composite, bridging cracks and delaying their propagation under functional loads (69, 73). Consequently, their incorporation may extend the longevity of restorations and facilitate future repair rather than replacement in case of failure. This characteristic represents a significant clinical advantage, particularly in minimally invasive restorative dentistry, where preserving as much sound tooth structure as possible remains a primary goal (72).

The finding that polyethylene fibers reduce the risk of unfavorable failures must be interpreted with consideration of an important methodological limitation. The criteria for classifying a failure as “catastrophic” or “favorable” were not standardized across the included studies. While some studies used strictly anatomical criteria (e.g., root fracture = catastrophic), others incorporated reparability. Therefore, the pooled risk difference from the meta-analysis does not represent a single, universally defined outcome but an aggregate of each study's own classification. Despite this limitation, the consistent direction of effect across all studies is notable and aligns with the proposed mechanical mechanism of fibers: by bridging cracks and distributing stress, they appear to **redirect failure towards patterns that are more likely to be confined to the coronal restoration**, as perceived by different research groups. Future *in vitro* studies would greatly benefit from adopting a common, clinically relevant failure classification system to allow for more robust quantitative synthesis.

Despite the consistent findings of this meta-analysis, several limitations should be acknowledged. First, only *in vitro* studies were included, which do not fully reproduce the complex clinical conditions of the oral environment, such as humidity, masticatory dynamics, and thermal fluctuations. Second, most of the included studies presented a medium to high risk of bias due to the absence of blinding and sample size calculation. Also, the lack of standardized testing protocols and long-term fatigue loading models limits the extrapolation of these results to clinical performance. Furthermore, the evidence synthesized is almost exclusively based on one commercial brand of polyethylene fibers (Ribbond™). While this reflects the focus of the existing *in vitro* literature, it limits the generalizability of our conclusions to other polyethylene fiber systems, which may differ in fiber architecture, surface treatment, and mechanical properties.

Worth is mentioning that our analyses revealed high statistical heterogeneity, which is a major limitation. This heterogeneity is an inherent feature of the *in vitro* literature in this field, stemming from substantial variations in study methodologies. This limitation underscores that our findings represent an average effect across a wide range of experimental conditions and highlights the need for more standardized reporting in future *in vitro* research. The high statistical heterogeneity precludes a precise, single estimate of the effect size of polyethylene fiber reinforcement. However, this variability itself is clinically informative. It reflects the reality that the restorative outcome is influenced by multiple factors at the chairside. The positive and significant pooled effect across such a spectrum of *in vitro* conditions—different cavity types, tooth states, and material combinations—is a robust indicator of a fundamental reinforcing capacity. For the clinician, this suggests that incorporating polyethylene fibers is a sound strategy to generally increase fracture resistance. Crucially, the heterogeneity signals that the *degree* of improvement may vary. For instance, a severely compromised endodontically treated molar with an MOD cavity may experience a more pronounced benefit from strategic fiber reinforcement than a vital premolar with a conservative restoration. Thus, the review's findings validate the reinforcement concept while simultaneously emphasizing that its execution—guided by principles of biomechanics and adhesive dentistry—is key to harnessing its full potential.

## 5 Conclusion

In conclusion, this study demonstrates that polyethylene fiber reinforcement can significantly enhance the fracture resistance of permanent teeth, but this effect is modulated by technical factors. Clinicians should be aware that its successful application depends on adherence to techniques that ensure optimal fiber integration and orientation. Moreover, in the included *in vitro* models, the use of polyethylene fibers was associated with a significant reduction in failures classified as catastrophic by the investigators. This suggests a favorable shift in failure pattern under laboratory conditions, which, if replicated *in vivo*, could potentially influence the clinical reparability of restored teeth. The clinical implications of this failure mode shift remain to be investigated.

## Data Availability

The raw data supporting the conclusions of this article will be made available by the authors, without undue reservation.
