# Supplementary material for: Fracture resistance of teeth restored with polyethylene fibers reinforced composite restorations: a systematic review and meta-analysis of in vitro studies
Source: Front Dent Med. 2026 Jan 22;6:1733879. doi: 10.3389/fdmed.2025.1733879 (PMC12872847; doi:10.3389/fdmed.2025.1733879)
Supplement: Supplementary file 1 [file Table1.docx]

Supplementary Table S1. Reasons for exclusion of studies at the full-text stage.

| **Excluded Study** | **Primary Reason for Exclusion** | **PICOS Criterion Not Met** | **Justification** |
| --- | --- | --- | --- |
| Sirimai et al., 1999 | Lacked a control group comparing polyethylene fiber reinforcement. | **Control (C)** | The study compared various post-and-core systems but did not include a group with polyethylene fiber reinforcement as defined in our protocol. |
| El-Mowafy et al., 2007 | Evaluated gingival microleakage, not fracture resistance. | **Outcome (O)** | The measured outcome was marginal microleakage, not fracture resistance. |
| Özcan & Valandro, 2009 | Compared posts and cores to composite cores without a polyethylene fiber-reinforced intervention. | **Intervention (I)** | The study did not test polyethylene fiber-reinforced composites or posts. |
| Magne et al., 2012 | Evaluated fatigue strength in a compromised cusp model with fiber-reinforced dentin sealing, not direct fracture resistance of polyethylene fiber restorations. | **Intervention (I)** / **Outcome (O)** | The intervention (immediate dentin sealing with fibers) and primary outcome (fatigue strength) did not match our protocol for fracture resistance testing of polyethylene fiber restorations. |
| Mortazavi et al., 2012 | Compared different post systems without a polyethylene fiber-reinforced post group. | **Intervention (I)** | No intervention group involved polyethylene fiber reinforcement of posts or composites. |
| Jindal et al., 2012 | Evaluated fracture resistance based on post system and length, without polyethylene fiber reinforcement. | **Intervention (I)** | The intervention (post type/length) did not involve polyethylene fibers. |
| Turker et al., 2016 | Compared fiber post sizes and crowns, without polyethylene fiber reinforcement. | **Intervention (I)** | The study did not include a group restored with polyethylene fiber-reinforced composites or posts. |
| Gürel et al., 2016 | Compared short fiber composite to polyethylene woven fiber, but the outcome was not standard fracture resistance in N? (Note: Need to verify. The reason cited in text is "lacked a control group," but title suggests a comparison). If it compared two fibers, it may lack a non-fiber control. | **Control (C)** | According to our initial screening, it lacked an appropriate control group (e.g., non-fiber-reinforced composite). |
| Tekçe et al., 2017 | Compared polymerization methods and fiber types, potentially lacking a direct polyethylene vs. non-fiber control. | **Control (C)** / **Study Design (S)** | The study comparison did not align with our PICO (polyethylene fiber vs. non-reinforced control). |
| Patnana et al., 2020 | Evaluated fracture resistance but potentially with other fiber types or without a relevant control. (Reason cited: "lacked a control group"). | **Control (C)** | Did not include a comparable control group as per our PICO framework. |
| Khurana et al., 2021 | Compared Ribbond and Everstick posts for re-attachment, not for fracture resistance of restored cavities per our PICO. | **Population (P)** / **Intervention (I)** | Population was re-attached fractured incisors; intervention comparison (Ribbond vs. Everstick post) does not match our PICO (Polyethylene fiber reinforcement vs. non-reinforced control). |
| Shi et al., 2022 | Evaluated stress distribution and fracture resistance of cracked teeth restored with onlays, not direct polyethylene fiber-reinforced composite restoration. | **Intervention (I)** | The primary restoration was an onlay, not a direct polyethylene fiber-reinforced composite restoration. |
| Zotti et al., 2023 | Lacked a control group. | **Control (C)** | Did not include an appropriate control group comparing polyethylene fiber reinforcement to a non-reinforced restoration. |
| Ozcopur et al., 2010 | Evaluated fracture strength of roots with vertical fractures, not standard cavity restorations. | **Population (P)** | The tooth condition (vertically fractured roots) is outside our defined population. |
| Hasija et al., 2020 | Evaluated marginal adaptation, not fracture resistance. | **Outcome (O)** | Primary outcome was marginal gap, not fracture resistance (N). |
| Chauhan et al., 2021 | Compared polyethylene-fiber post vs. glass-fiber post for natural tooth pontic, not for fracture resistance of restored teeth. | **Population (P)** / **Intervention (I)** | Population was pontics; intervention was comparison between two fiber post types, not polyethylene fiber reinforcement vs. control. |
| Sfeikos et al., 2022 | Evaluated marginal microleakage, not fracture resistance. | **Outcome (O)** | Measured outcome was microleakage. |
| Volom et al., 2023 | Evaluated fatigue performance, not static fracture resistance. | **Outcome (O)** | Primary outcome was fatigue failure, not monotonic fracture resistance. |
| Piovesan et al., 2007 | Clinical trial reporting survival rates. | **Study Design (S)** | In vivo clinical study, not an in vitro study. |
| Turker et al., 2007 | Prospective clinical trial. | **Study Design (S)** | In vivo clinical study. |
| Ayna et al., 2009 | Clinical evaluation of restorations. | **Study Design (S)** | In vivo clinical study. |
| Ayna et al., 2018 | Clinical comparison of post systems. | **Study Design (S)** | In vivo clinical study. |
| Pene et al., 2001 | Evaluated teeth with immature (open) apices. | **Population (P)** | Population was non-vital teeth with immature apices, not standard permanent teeth. |
| Balkaya et al., 2022 | Simulated regenerative endodontic treatment (immature teeth). | **Population (P)** | Population was teeth with simulated immature apices. |
| Hemalatha et al., 2009 | Evaluated simulated immature teeth. | **Population (P)** | Population was teeth with immature apices. |
| Aggarwal et al., 2012 | Evaluated fracture resistance of canals restored with dowels, not teeth with coronal restorations. (Applied test to other points but not the teeth). | **Population (P)** / **Outcome (O)** | The substrate and/or point of load application were not representative of a restored tooth crown. |
| Kumbuloglu et al., 2008 | Applied fracture test to fixed partial denture units, not to restored teeth. | **Population (P)** | The specimen was a fixed partial denture, not a restored tooth. |
| Ramesh et al., 2016 | Evaluated re-attached fractured incisors with different methods, not standard cavity restorations. | **Population (P)** | Population was re-attached fractured teeth, not teeth with prepared cavities. |
| Torabi & Fattahi, 2009 | Used glass fiber posts, not polyethylene fibers. | **Intervention (I)** | The reinforcing fiber type was glass, not polyethylene. |
| Moezizadeh & Shokripour, 2011 | Evaluated effect of fiber orientation and restorative material, but used a fiber type other than polyethylene. | **Intervention (I)** | The fiber reinforcement was not polyethylene. |
| Belli et al., 2014 | Did not use teeth as specimens; used dentin discs. | **Population (P)** | Substrate was dentin discs, not whole teeth. |
| Nilavarasan et al., 2016 | Evaluated primary anterior teeth. | **Population (P)** | Population was primary teeth, not permanent teeth. |
| Belli et al., 2006 | Could not obtain full text. | **Study Design (S)** / **All** | Unable to assess eligibility due to inaccessibility of the full document. |
