# Supplementary figures and images for: Fracture resistance of teeth restored with polyethylene fibers reinforced composite restorations: a systematic review and meta-analysis of in vitro studies
Source: Front Dent Med. 2026 Jan 22;6:1733879. doi: 10.3389/fdmed.2025.1733879 (PMC12872847; doi:10.3389/fdmed.2025.1733879)

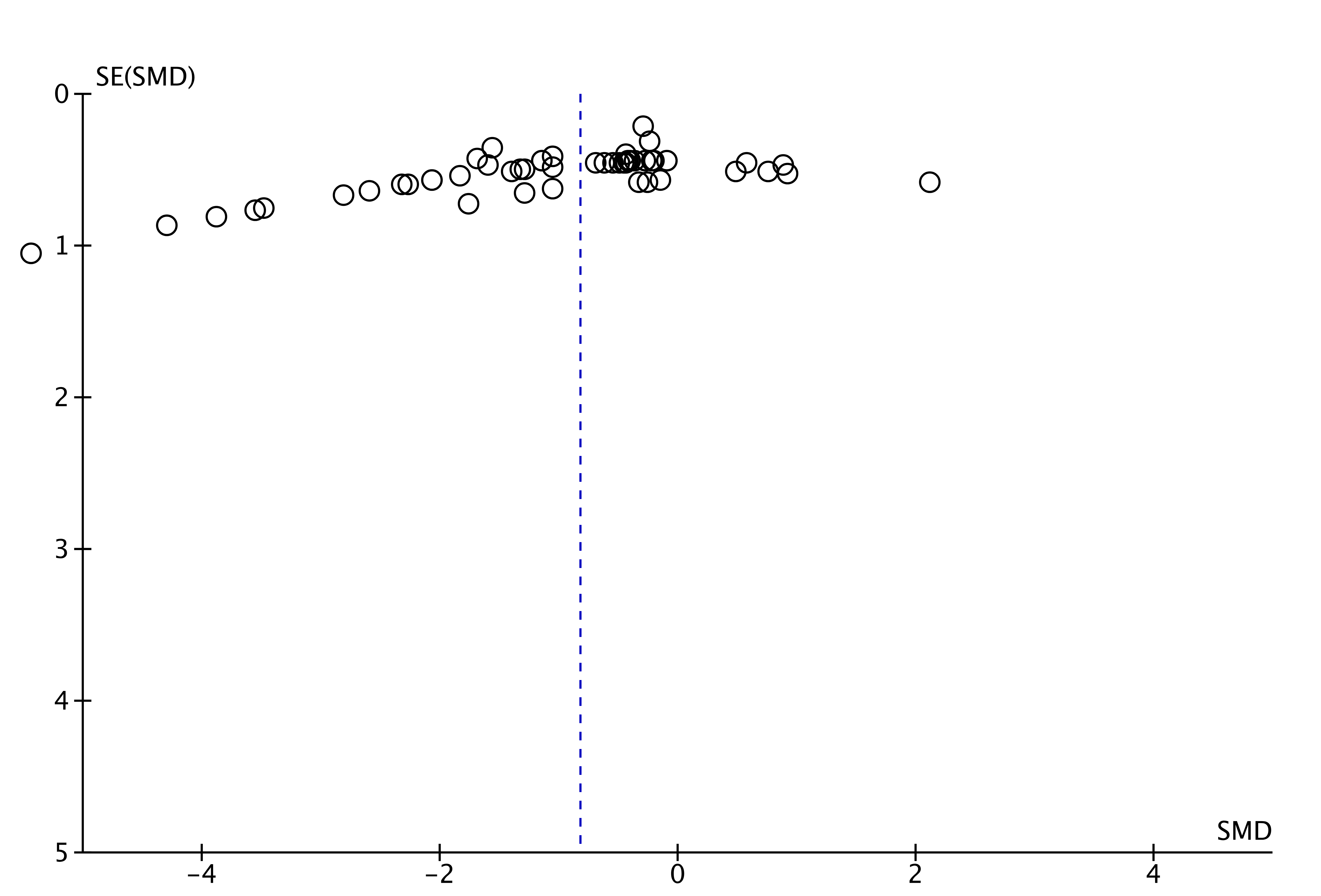

Supplement: Supplementary file 2 [file Image2.tiff]

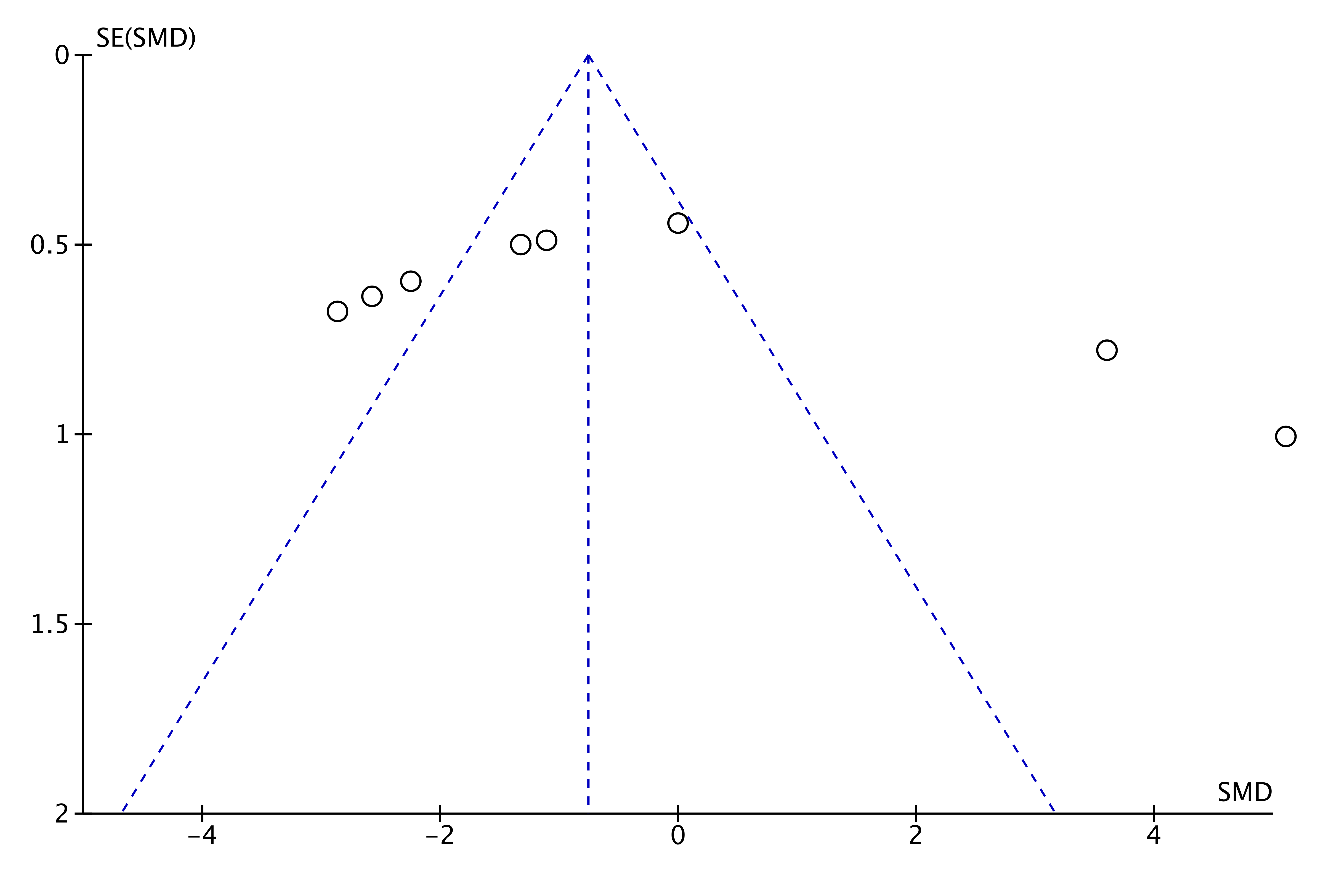

Supplement: Supplementary file 3 [file Image1.tiff]

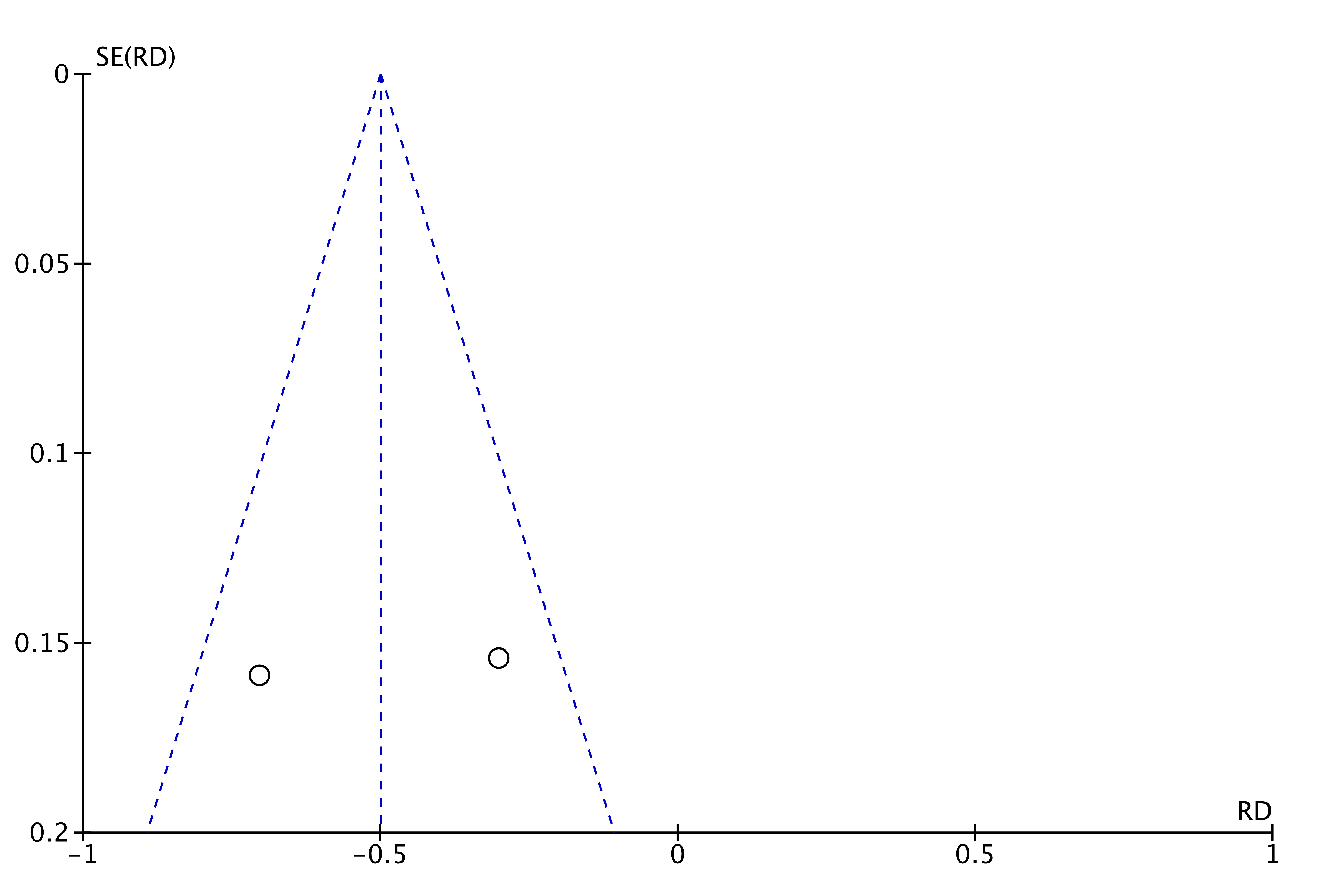

Supplement: Supplementary file 4 [file Image3.tiff]

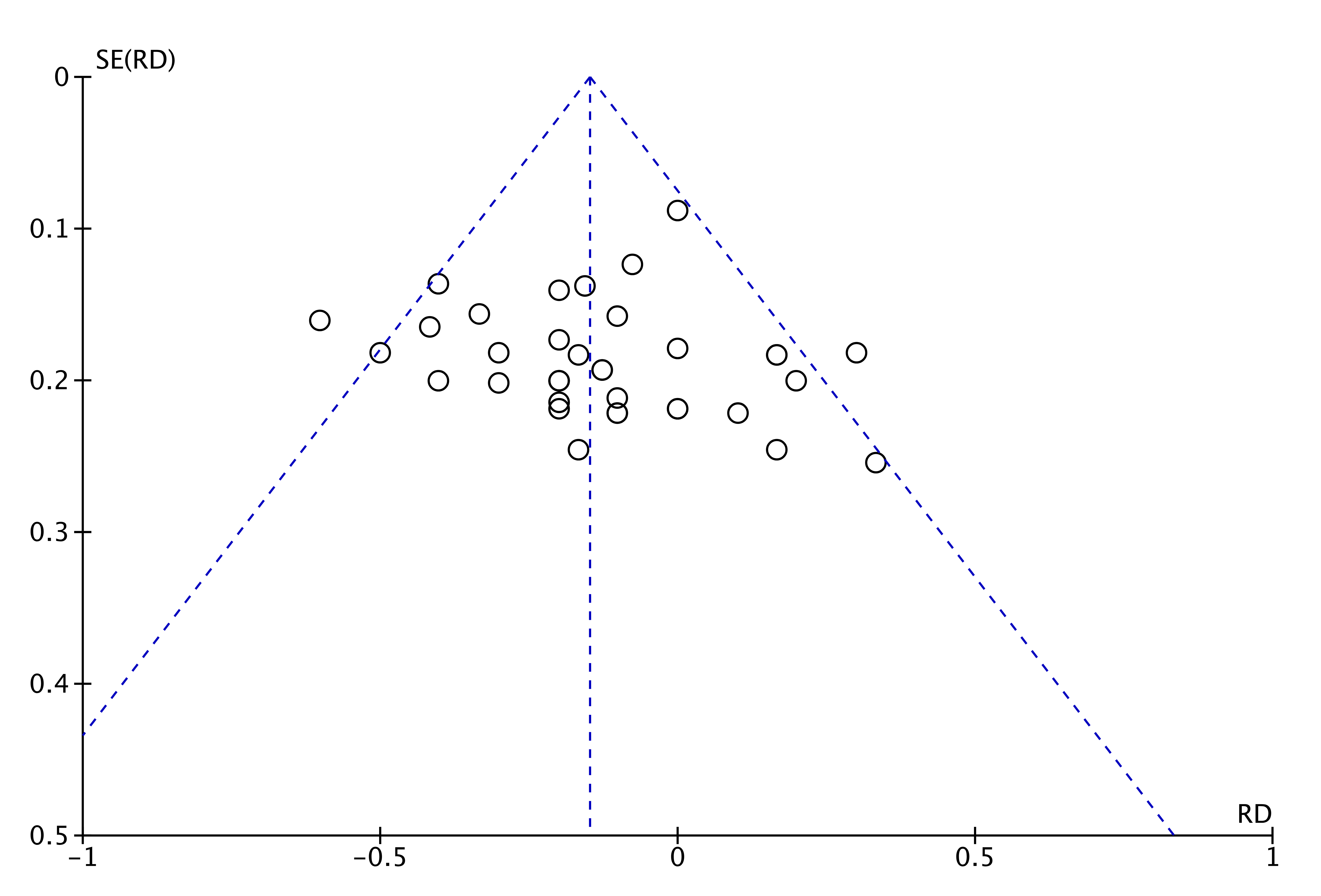

Supplement: Supplementary file 5 [file Image4.tiff]
